# Non-Linear Biomechanical Evaluation and Comparison in the Assessment of Three Different Piece Dental Implant Systems for the Molar Region: A Finite Element Study

**DOI:** 10.3390/jfb16010017

**Published:** 2025-01-09

**Authors:** Jesus Alejandro Serrato-Pedrosa, Ignacio Villanueva-Fierro, Rodrigo Arturo Marquet-Rivera, Rosa Alicia Hernández-Vázquez, Salvador Cruz-Lopez, Verónica Loera-Castañeda

**Affiliations:** 1 CIIDIR-Durango, Instituto Politécnico Nacional, Calle Sigma 119, Fraccionamiento 20 de Noviembre II, Durango C.P. 34220, Mexico; ivillanuevaf@ipn.mx; 2 Escuela Superior de Ingeniería Mecánica y Eléctrica Unidad Culhuacán, Instituto Politécnico Nacional, Av. Sta. Ana 1000, San Francisco Culhuacán, Colonia Culhuacán CTM V, Alcaldía Coyoacán, Ciudad de México C.P. 04440, Mexico; rmarquetr@ipn.mx; 3 Escuela Superior de Comercio y Administración Unidad Tepepan, Instituto Politécnico Nacional, Anillo Periférico Sur Manuel Gómez Morín 4863, Colonia Ampliación Tepepan, Alcaldía Coyoacán, Ciudad de México C.P. 16020, Mexico; 4 División de Mecatrónica, Universidad Politécnica del Valle de México, Av. Mexiquense s/n esquina Av. Universidad Politécnica, Col. Villa Esmeralda, Tultitlán C.P. 54910, Estado de México, Mexico; alicia.hernandez@upvm.edu.mx; 5 MARMAS Soluciones en Ingeniería e Investigación, Matagalpa 1021A, Residencial Zacatenco, Gustavo A. Madero, Ciudad de México C.P. 07369, Mexico; scruzl@marmas-soluciones.com

**Keywords:** dental implant systems, finite element method, numerical approach, mechano-biological behavior, biomechanics

## Abstract

The widely available options of different manufacturers in dental implant systems have complicated the selection criteria process for periodontists, necessitating careful consideration of various factors when selecting suitable solutions for individual patient needs. Optimal implant selection requires careful consideration of the patient-specific factors, implant design, and surgical technique. Understanding the biomechanical behavior of implant–tissue interactions is crucial for achieving successful and long-lasting implant therapy. To adequately address this issue and improve the rigorous selection criteria from a biomechanically numerical approach, this research aims to analyze the stress distribution fields, strain patterns, and load transfer displacements within the implant system and the implant–biological interface (gingival and bony tissues) of titanium three-piece to two–one-piece ceramic implant systems. Thus, three different commercially available dental implants designed to be placed in the jaw molar region were considered for evaluation through the finite element method under both oblique and occlusal loading conditions. The results have exhibited an increasing trend to highlight the outstanding behavior of two-piece ceramic implants to dissipate the stress distribution better (6 and 2 times lower than the three- and one-piece systems under occlusal loads and almost 5 and 1.3 times more efficient for oblique loading, respectively), minimize peak stress values (below 100 MPa), and reduce strain peak patterns compared with the other two evaluated designs. On the other hand, the effects generated in biological tissues are strongly associated with implant geometry features. This biomechanical approach could provide a promising strategy for predicting micro-strains and micromotion in implant system pieces and geometries. Hence, these findings contribute to a deeper understanding of the biomechanics spectrum in the behavior of dental implant systems and emphasize the importance of carefully selecting appropriate material systems for accurate patient-specific biomechanical performance.

## 1. Introduction

Dental implant design has been evolving with significant advancements for optimizing clinical assessments. In this regard, dental raw restoration has evolved, with solutions ranging from three- to one-piece implants. One-piece implants, characterized by their integrated design, offer a potential advantage by eliminating the micro-gap often present in two- and three-piece implants. Their integrated structure combines the prosthetic component and surgical unit into a single entity. This design ensures continuous transmucosal presence, enhancing the potential for optimal tissue integration [1,2]. Two- and three-piece implants, with their separate abutment and implant body, may introduce a micro-gap at the junction; in addition, material conditions are a relevant factor in micromovements generation due to the diminutive gap in titanium and cobalt–chrome three-piece implants (mostly metal implants) [3,4].

Nowadays, the wide availability of dental implant systems has presented dentists with a complex decision-making process when selecting appropriate options for diverse clinical scenarios, having a myriad of variables to select from for an adequate type of system implant [5]. In general, specialists base their selection criteria on a common critical factor, which is the implant–abutment interaction behavior [6].

The recent literature and meta-analyses have revealed interesting findings in the clinical performance of marginal bone loss. Although all of the implant system types exhibit different behaviors, all of the implants are constant in marginal bone loss and survival rates [7,8]; in this sense, preliminary evidence suggests that in two-piece implant systems, abutments exceeding 2 mm in height might potentially mitigate alterations at the marginal bone level [9]. Likewise, clinical analyses comparing one-piece and two-piece ceramic implants indicate better versatility in two-piece implants compared with single units. Nonetheless, further comprehensive enduring scientific data are necessary to validate their superior applicability over one-piece implants [10]; as current clinical evidence for two-piece ceramic implants remains limited, particularly regarding long-term outcomes, with a minimum follow-up period of five years, the implants must be properly informed to patients before prosthesis placement [11].

In order to advance scientific-based clinical practice and optimize patient outcomes in dental implantology, further research is imperative to comprehend the underlying mechanisms governing peri-implant bone adaptation and the long-term durability of dental implants [12]. Accordingly, biomechanical analyses of dental implant–piece configurations under functional loading conditions are essential. A primary focus of research has been on analyzing the behavior between the implant, abutment, and screw components, where the principal objective is to maintain stress fields below the failure stress of the bone [13,14,15]. To evaluate the performance of dental implants and ensure long-term success, it is essential to understand the distribution of forces during mastication [16]. The implementation of finite element analysis/method (FEA/FEM) employs numerical estimation techniques to mechano-biologically predict the stress distribution within the implant–bone interface [17,18], providing valuable insights into the influence of prosthetic biomechanical properties on dental implant performance, and consequently assess the impact of force distribution on treatment outcomes [19,20,21]. Adaptive models of bone tissue and mathematical material models play a crucial role in understanding the complex behaviors of bone in response to mechanical stimuli and the then-triggered biological effects. Bone tissue is a structure that continuously undergoes remodeling, characterized by the coordinated activity of bone cells that remove old bone tissue and generate new bone tissue [22,23]. External agents are transmitted in stress and strain distribution fields to osteocytes; these stimuli secrete signals that stimulate mononuclear cell proliferation, leading to osteoclast and osteoblast activity [24]. Thus, developing adaptive models facilitates numerical simulations of these complex remodeling processes, providing insights into bone health and disease. Mathematical material models are crucial in understanding and simulating the complex processes involved in bone tissue behavior and healing. Mechanical strain has an outstanding performance in maintaining bone density and structural integrity; this adaptive process parameter is governed by mechanobiological principles, where the application of load induces cellular responses that can either promote bone formation or resorption [25,26]. Particularly, employing the finite element method can permit the analysis of bone behavior under varying mechanical conditions, facilitating a deeper understanding of the biomechanics involved. For instance, recent studies have presented an adaptation model based on mechanical levels, with simulations being conducted under different loading conditions to assess how bone architecture responds to these changes [27]. The total mechanical stimulus at any location on the bone surface is determined by the contributions from all osteocytes relative to their distance from that point.

Therefore, the current research work has the main aim of thoroughly analyzing the stress distribution and strain effects in three designs of different implant system types based on clinically available solutions in the market. Such implant systems are designed to be placed in any of the three molar regions of the jaw with the same rehabilitation applications (low bone density, immediate loading performance). Thus, the employment of numerical simulations remains crucial for deepening the comprehension of micro-gaps between elements, implant–abutment contact, and implant–surrounding bone biomechanical behaviors. Furthermore, mechanically analyzing dental implant systems through finite element analyses promotes personalized implant design and optimizing placement strategies in medical assessment.

## 2. Materials and Methods

A finite element analysis was conducted to compare three different implant systems based on the manufacturers’ designs for placement in the molar region and their interaction with three biological tissues: cortical bone, trabecular bone, and gingival tissue. The primary objective was to evaluate the behavior of the stress distribution fields and strains generated within the body of the dental implant, biological tissues, and implant–abutment interaction. Furthermore, a discretization convergence analysis was performed to assess the accuracy and efficiency of the numerical model. Hence, smaller sizes are provided for crucial zones of the dental implant threads and the biological threaded zones.

### 2.1. Modeling of Three-Dimensional Implant Systems

**Figure 1:**
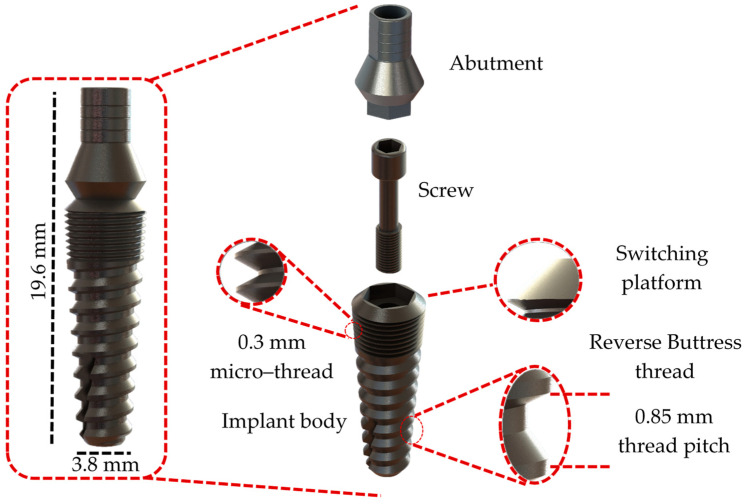
CAD modeling of the three-piece implant system design.

**Figure 2:**
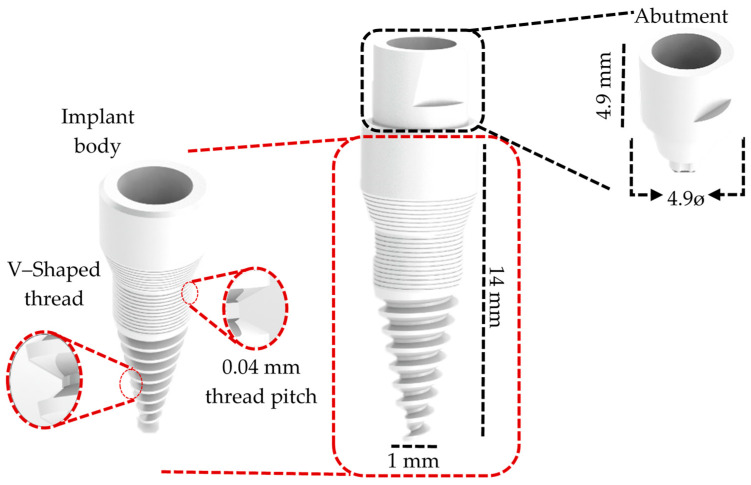
Three-dimensional two-piece implant system design.

**Figure 3:**
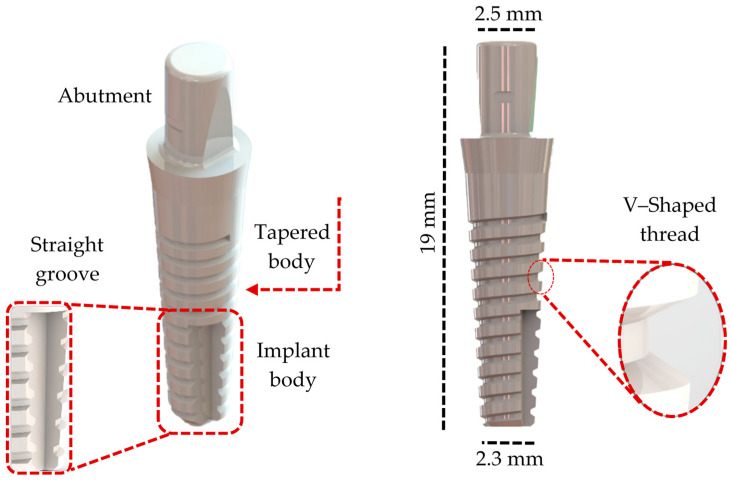
One-piece implant system generated model.

The 3D modeling phase of the three dental implant models was developed to reproduce a clinical situation, replace an implant in the jaw molar regions, and simulate functional occlusal loading on systems with different numbers of implant pieces. The three-piece implant system was developed using the literature and catalogs of the used brand (IML SA Swiss Dental Implants, Mendrisio, TI, Switzerland) through a reverse engineering process and utilizing the titanium GR.4 Infinity Implant System as a reference (available in INFINITY Ed. 2023_rev 1 catalog) [28]. The geometric design of the implant body is a well-suited solution for low bone density (Figure 1). Ceramic solutions were chosen for the two-piece and single-unit implant systems. Particularly, the two-piece system was based on the TZP-A (Tetragonal Zirconia Polycrystal) SDS2.2 dental implant system (SDS SWISS DENTAL SOLUTIONS AG, Kreuzlingen, TG, Switzerland; available in 2024 product catalog SDS1.2 and SDS2.2) [29] (Figure 2). Consequently, the one-piece implant design was based on the Tree·Oss^®^ Ceramic one-piece zirconia implant (Tree·Oss^®^, Ensenada, BA, Argentina; available in Tree·Oss 2023 catalog) [30] (Figure 3). Furthermore, all implant system models were modeled using the computer-aided design (CAD) software Solidworks2023^®^. Moreover, the selection criteria for implant system configurations were based mainly on their function, applicability, implant length, and wide usage in the mandibular molar region in commercially available solutions in the 2024 and 2020s catalogs. It is important to note that the primary objective of this study was to compare the mechanobiological behavior of different elements in dental implant systems under similar loading conditions. Selecting different materials (ceramics and metal) and geometries as the main criteria can generate a wider spectrum in the factors, influencing stress distribution, strain patterns, and the overall implant stability of commercial designs. For instance, selecting a smaller one-piece implant system was intended to analyze the impact of design variations on stress distribution and bone strain while acknowledging the limitations imposed by the available implant sizes. Molar teeth were selected for this study due to their complex anatomical structure and due to the significant functional demands placed upon them during mastication (complex loading conditions). Given the prevalence of molar tooth loss and the challenges associated with their replacement, specifically, the first molar was selected as the focus of this investigation due to its unique anatomical position, experiencing a combination of occlusal and oblique forces during mastication, and its frequent loss due to various factors such as caries, periodontal disease, or trauma. Specific studies have stated that the first molar is the most commonly replaced tooth with dental implants. Also, the dental literature and clinical practice have supported this assertion [31]. For each experimental group, different implant systems from various commercial manufacturing companies that offered multiple-piece dental systems (from one to three-piece solutions) were analyzed before selecting the ones evaluated.

**Figure 4:**
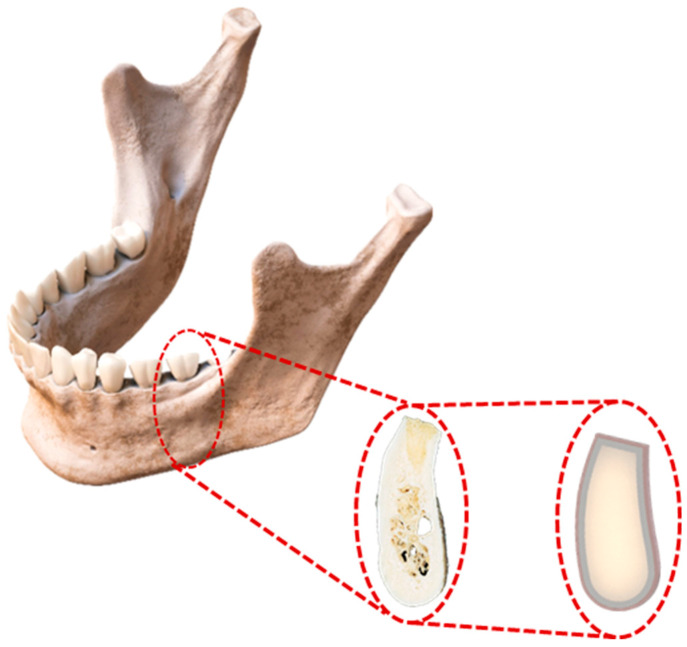
Schematic view of the 3D mandibular section block.

To adequately analyze the behavior of the surrounding biological tissues, the morphology of the molar region was generated from a cross-section view of the mandible, representing a block of mandibular tissue simulating cortical and trabecular bones along the gingiva. The block length is 21 mm with a 5.5 mm thickness, corresponding to average jaw dimensions and suitably aligning with the designed block measurements [32] (Figure 4). Bone structures were considered to have an average proportion of cortical and cancellous bones of approximately 80% and 20%, corresponding to Type 2 bone density (healthy bone) according to the Lekholm and Zarb classification [33] or D2 bone density classification by Mish (average optical density of 850–1250 Hounsfield units for the mandible) [34].

Once the geometries of the implant systems were fully designed, the finite element analysis software ANSYS Workbench^®^ 2021 R2 was employed to establish the simulation parameters. The type of mechanical analysis, assignment of the biomechanical properties to the relevant structures, discretization of the geometries, and appropriate boundary conditions and external loads were applied.

### 2.2. Finite Element Discretization Process

**Figure 5:**
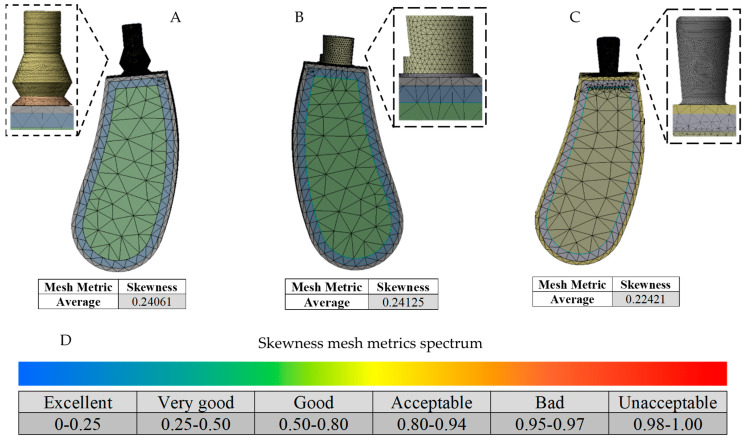
Discretization process and mesh quality testing. (**A**) Three-implant system discretization and zoom-in view of the finer mesh. (**B**) Two-implant system discretization and zoom-in view of the finer mesh. (**C**) One-implant system discretization and zoom-in view of the finer mesh. (**D**) Ansys skewness mesh metrics spectrum.

The discretization process was generated by employing high-order 3D solid elements (Solid187) having 20 nodes per element (tetrahedral elements). A discretization convergence analysis was performed to evaluate the sensitivity of the numerical results to the size and distribution of the finite elements used in the simulations; it was set at a change in the displacement variation of less than 1% for the model with different element sizes. Thus, an adaptative mesh refinement was developed [35,36,37], with the element size being controlled to approximately 0.1 mm in the zones of interest and the implant threads, abutment, and the biological tissue threaded part being controlled to 1 mm (Figure 5).

The results show that the size and distribution of the finite elements are important factors influencing the accuracy of the results. To ensure a high-quality mesh for all analyses, the corresponding tetrahedral element meshing method parameter was employed (skewness mesh metrics spectrum) [38,39], having an average of 0.24061 for the three-implant system simulation, 0.24125 in the two-implant configuration, and 0.22421 in the single-implant system; this corresponds to excellent mesh quality according to ANSYS Workbench^®^ mesh metrics recommendations. Table 1 details the elements and nodes utilized in each implant system analysis.

### 2.3. Loading and Boundary Conditions

**Figure 6:**
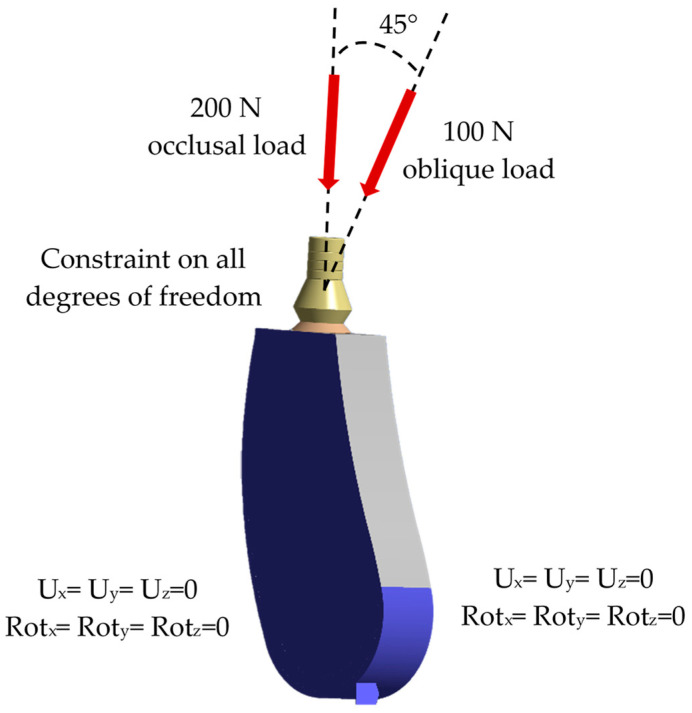
Loading and boundary condition for all dental implant systems.

Suitable boundary conditions were assigned to simulate realistic clinical conditions and maintain mechanical equilibrium. All implant systems were completely constrained in the bone’s side surfaces and the bottom part of the block zero degrees of freedom (Ux = Uy = Uz = 0) and rotations (Rotx = Roty = Rotz = 0) in all directions. The assignment of the external agent was represented by simulating the bite force condition (vertical occlusal and oblique effect), described as a complex loading condition (normal and tangential forces). Hence, 200 N of occlusal and 100 N of oblique loads were applied to models to replicate a real estimate behavior according to the literature [40,41,42,43] (Figure 6). Contact between elements was represented through contact surfaces using 8-node 3D surface-to-surface contact elements CONTA174 and their corresponding target element TARGE170. The bone–implant and bone–bone interfaces were assumed to be perfectly bonded, simulating ideal osseointegration with complete contact between the implant and the surrounding bone tissue, as well as cemented fixed conditions for the two-piece implant.

### 2.4. Mechanical Properties of the Elements

The assignment of the mechanical properties corresponds to each material utilized in the simulation. In order to simulate a closer-to-reality behavior, bony tissue is considered as an orthotropic linear material (for both trabecular and cortical bone) [44,45,46]. The material properties of the dental implant components were assumed to be linear-elastic, continuous, homogeneous, and isotropic, using values reported in the literature [47,48,49,50]. A gingival tissue representation was added to the block to analyze a wider spectrum of mechanical behavior [51]. Table 2 summarizes the mechanical property values used in the study.

## 3. Results

### 3.1. Results of the Three-Implant System

**Figure 7:**
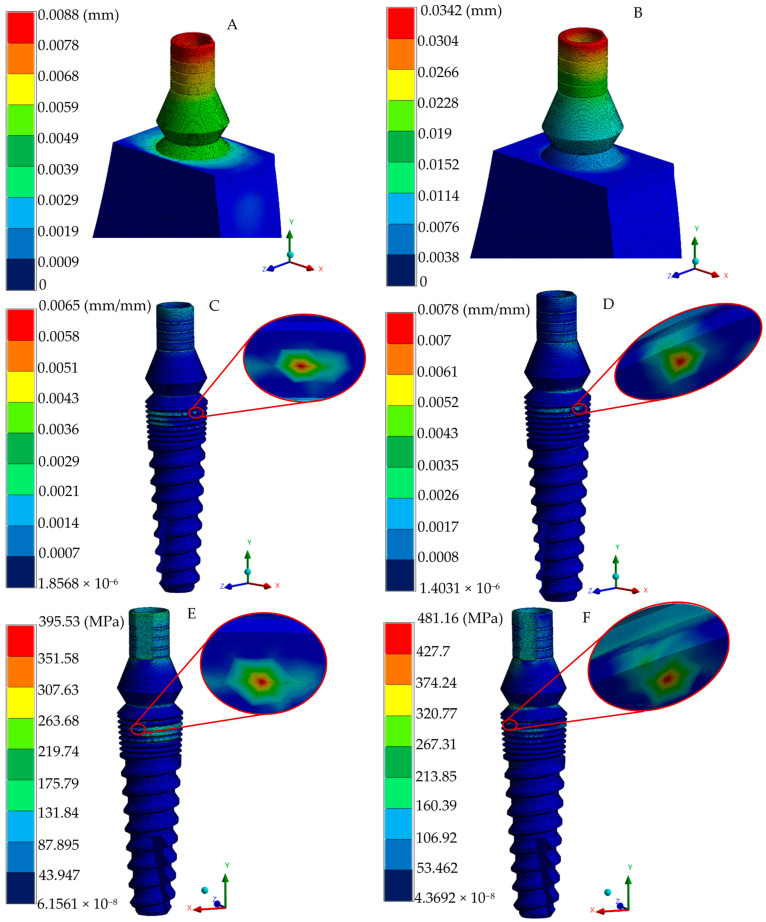
Implant numerical results of the three-piece system. (**A**) General displacement under occlusal loading. (**B**) General displacement under oblique loading. (**C**) Total elastic strain under occlusal loading. (**D**) Total elastic strain under oblique loading. (**E**) von Mises stress under occlusal loading. (**F**) von Mises stress under oblique loading.

**Figure 8:**
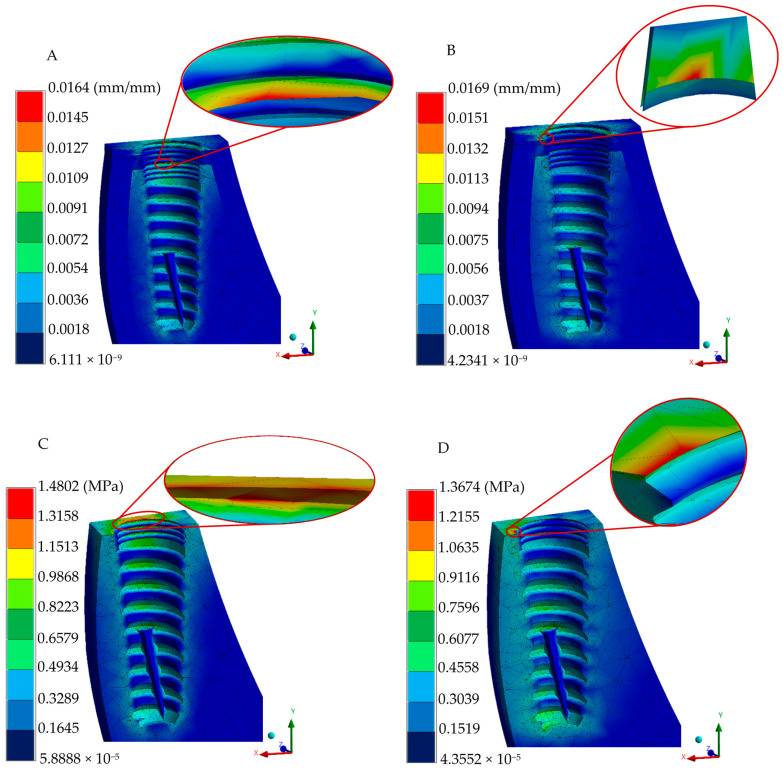
Biological structure numerical results of the three-piece system. (**A**) Total elastic strain under occlusal loading. (**B**) Total elastic strain under oblique loading. (**C**) von Mises stress under occlusal loading. (**D**) von Mises stress under oblique loading.

The adequate conditions generated in the discretization and loading boundary conditions (preprocessing stage) in the numerical analysis converged to provide accurate biomechanical predictions. In order to assess the complex stress states within the implant–bone interface, the von Mises stress failure theory was employed, enabling the evaluation of combined stresses from multiple directions. In addition, the implant and biological systems generate the displacements and strains. Figure 7 depicts displacements, strains, and the von Mises stress distribution for occlusal and oblique loading conditions in the implant, whereas Figure 8 shows the biomechanical behavior in the gingiva and bony tissues. Despite having an increment in peak values in oblique loading, distribution patterns quite were similar for the two loading types utilized.

### 3.2. Results of the Two-Implant System

**Figure 9:**
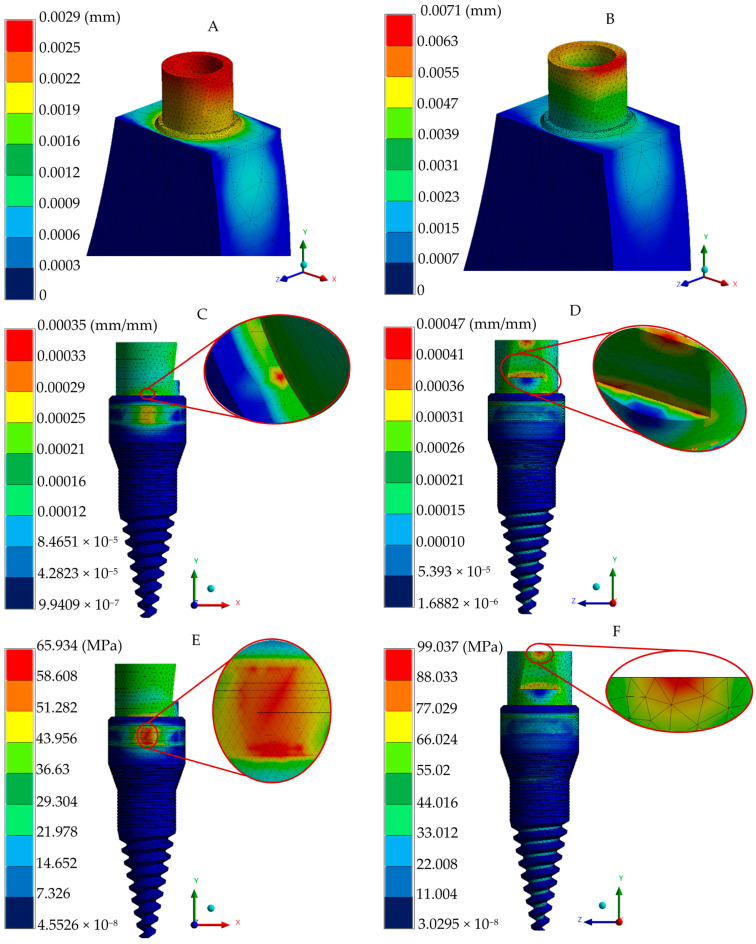
Implant numerical results of the two-piece system. (**A**) General displacement under occlusal loading. (**B**) General displacement under oblique loading. (**C**) Total elastic strain under occlusal loading. (**D**) Total elastic strain under oblique loading. (**E**) von Mises stress under occlusal loading. (**F**) von Mises stress under oblique loading.

**Figure 10:**
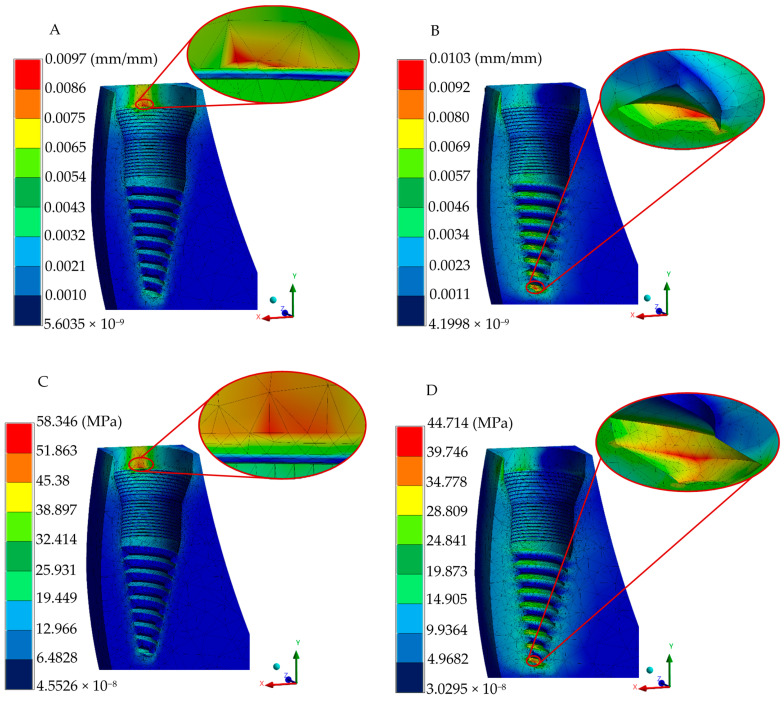
Biological structure numerical results of the two-piece system. (**A**) Total elastic strain under occlusal loading. (**B**) Total elastic strain under oblique loading. (**C**) von Mises stress under occlusal loading. (**D**) von Mises stress under oblique loading.

The resulting simulations for the two-implant system provided insights into the displacement, strain, and stress patterns in both the implant and surrounding tissues under loading conditions, as illustrated in Figure 9 and Figure 10. It is evident that the phenomenon occurred in oblique loading conditions to increase peak values but reduce the distribution fields; this can be attributed to a material change with better energy dissipation than titanium and zirconia (right-sided figures).

### 3.3. Results of the One-Implant System

**Figure 11:**
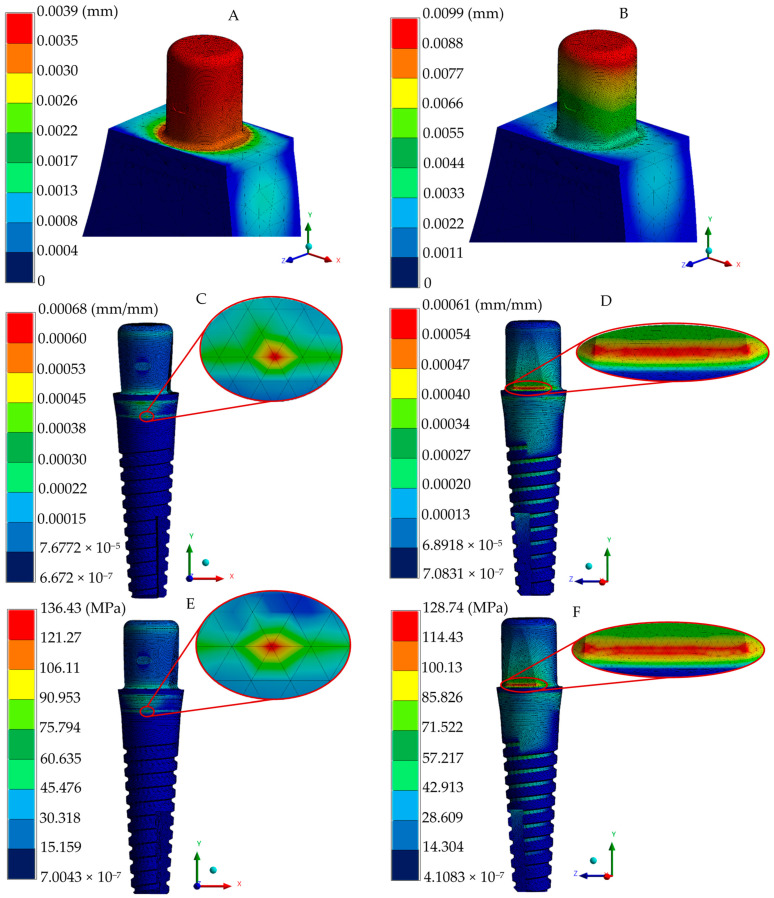
Implant numerical results of the one-piece system. (**A**) General displacement under occlusal loading. (**B**) General displacement under oblique loading. (**C**) Total elastic strain under occlusal loading. (**D**) Total elastic strain under oblique loading. (**E**) von Mises stress under occlusal loading. (**F**) von Mises stress under oblique loading.

**Figure 12:**
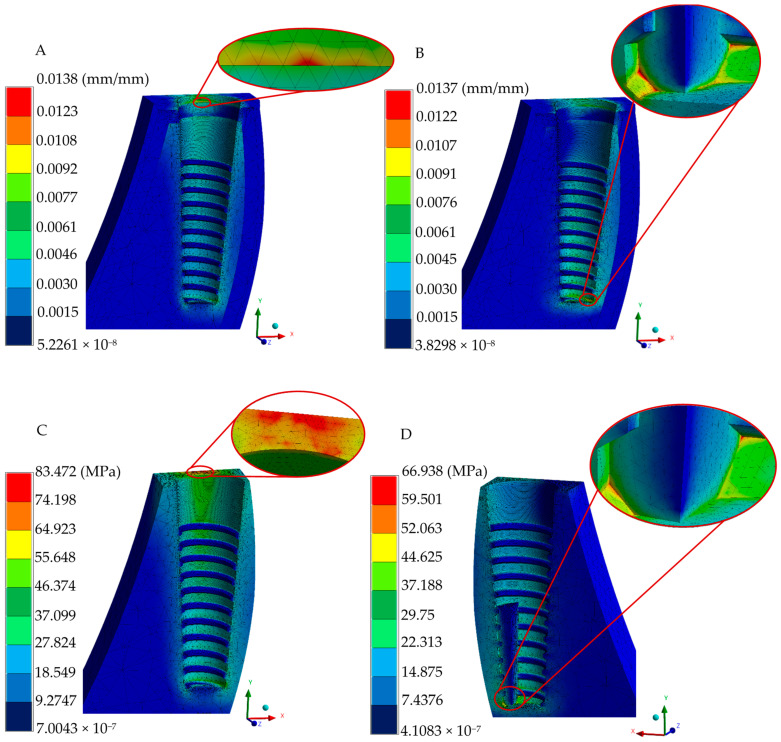
Biological structure numerical results of the one-piece system. (**A**) Total elastic strain under occlusal loading. (**B**) Total elastic strain under oblique loading. (**C**) von Mises stress under occlusal loading. (**D**) von Mises stress under oblique loading.

Figure 11 and Figure 12 illustrate the visual representations of the von Mises stress distribution, general displacements, and total strains in the single-unit implant. The isochromatic maps offer a visual representation of the regions subjected to peak values, facilitating the identification of potential failure sites and informing design characteristics in the mechanical performance of the implant system.

**Figure 13:**
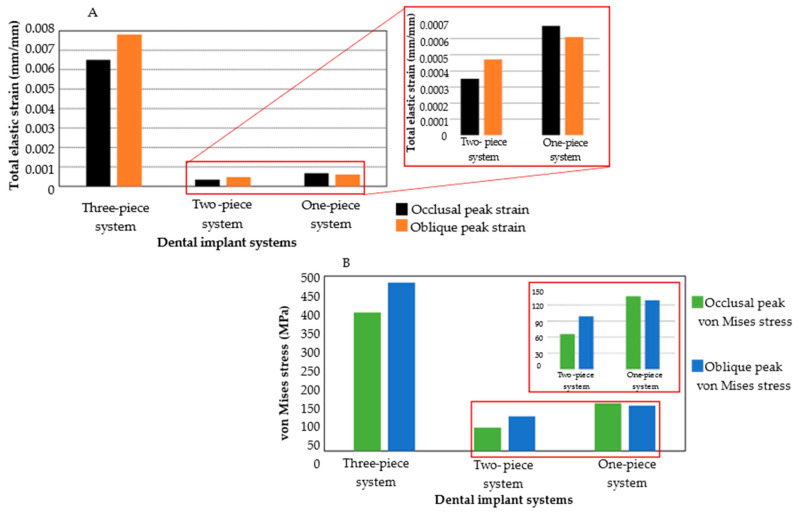
Comparison of numerical simulations for all dental implant systems. (**A**) Total elastic strain under both loading conditions. (**B**) von Mises stress under both loading conditions.

A comprehensive analysis of the stress and strain distribution within the system is necessary to assess the biomechanical impact of the implant systems on the surrounding bone tissue. Examining the areas of high and low stress concentration makes it possible to identify regions of potential overload or underload. A direct comparison in displacement results demonstrates a higher concentration in the one-piece implant due to being smaller than the other two designs. Bigger dimensions in the abutment of the two-implant system generate a smoother distribution, having lower peak values. Geometric and material properties are critical in the comparison criteria for assessing strain and stress distribution. The elastic and shear modulus of the ceramic implants is double that of the three-piece implant, which could lead to excessive strain distribution levels in the bone tissue that may develop micromotion and compromise the functional behavior of the implant system. Furthermore, in this regard, the zirconia mechanical properties, along with bigger and wider two-implant dimensions, demonstrate better dissipation of the overall stress state, where employing the von Mises stress failure theory predicted a yielding of the implant systems wherein the titanium implant had twice higher stress peak values than ceramic implants. Considering the highest strain value of the three-piece system, peak values were reduced in occlusal conditions by 18 times and 9 times for the two- and one-piece implant, respectively, along with a reduction of 16 and 12 times for oblique loading (Figure 13A). Likewise, peak stress values during occlusal loading were reduced five times and by two times for the ceramic implants (two and one-piece implants) compared with the three-piece dental implant. Meanwhile, during the oblique load application, the reduction was more significantly reduced for the one-piece implant by three times compared with the three-piece system, and the two-piece implant acquired a reduction by four times (Figure 13B).

**Figure 14:**
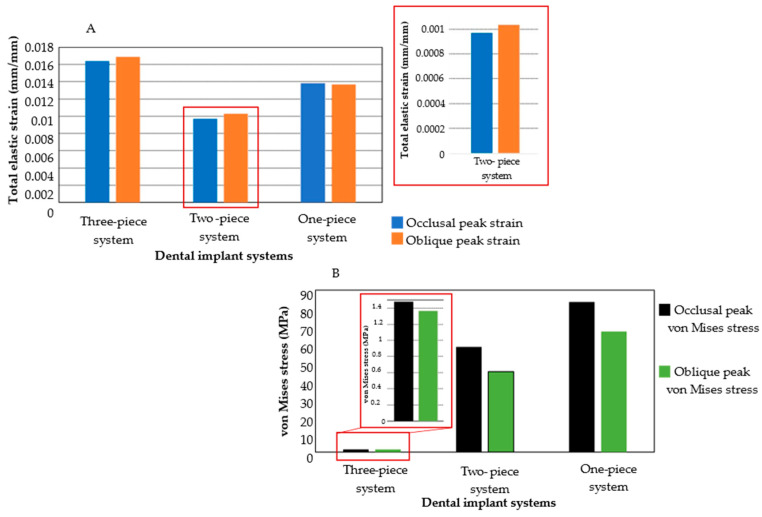
Comparison of numerical simulations for biological structures. (**A**) Total elastic strain under both loading conditions. (**B**) von Mises stress under both loading conditions.

When comparing the mechanobiological effects in bone and gingival structures, despite having different components in the two-implant system compared with the one-piece system, this implant system demonstrated the lowest strain values as having higher patterns near the implant–abutment joint (Figure 14A). The two-piece and three-piece implant systems exhibited similar distribution values and peak strain patterns under occlusal loads. On the other hand, under oblique loading conditions, strain peak values are located in the implant groove for the two- and three-implant systems. Stress concentration patterns in biological tissues are smaller for the three-piece implant system and higher in the one-piece system for the ceramics prosthesis (Figure 14B). Unlike the biomechanical behavior of the implant systems, oblique loading reduced peak stress and strain distribution fields in the biological tissues for all implant systems compared with occlusal application.

Selecting an orthotropic nature for bone structures becomes crucial when analyzing stress distribution and deformation under loading conditions. Bone’s anisotropic behavior can affect the propagation of stress distribution patterns and the overall mechanical response of the implant–bone interface. Therefore, while orthotropy is acknowledged as an important factor in bone mechanics for better replication, it is notable to mention that while orthotropy may not directly alter the yield stress and density values, it can significantly impact the overall mechanical behavior of the material along the three different tangential planes. In this regard, detailed numerical data along axis and tangential planes, including maximum and minimum values, are extended in Appendix A, Table A1, Table A2 and Table A3. Particularly in Table A1 for the three-piece implant system, the finite element results indicate an underload for the XZ tangential plane, whereas along the other two planes seemed to be physiological loading areas compared with the von Mises stress distribution for both the implant system and the assessment in biological tissues. The stress distribution within the YZ plane exhibited an area of overload contrasted to the low-stress values in the other two tangential planes for the two-implant system operation. Additionally, in the XY plane during oblique load, the peak stress value presented an overload in the one-piece implant system. In the XY plane, peak stress during occlusal loading presented the highest stress concentration for the implant system and jaw structure. Moreover, an underload behavior was exhibited in the XZ shear stress plane with minimum stress concentration values.

## 4. Discussion

The current research has been developed to provide insights into dentistry assessment from a numerical-biomechanical approach in a high-interest subject of criteria selection regarding implant systems configurations, commonly focused on implant–abutment interaction and materials [6,7]. A myriad of implant systems can be utilized for the same clinical applications from many commercial manufacturers; hence, the adequate evaluation of the whole spectrum of biomechanical behavior can pave the way for selecting the most suitable solution based on a deep understanding of the stress concentration fields, displacements, and strain distribution. Therefore, the methods applied align perfectly with what has been established in the literature, obtaining accurate results from quality mesh evaluation and proper assignment of the loading and boundary conditions. In this regard, results from this research illustrated a similar behavior in stress distribution patterns from previous studies when applying orthotropic properties to bone structures, which maximize the stress and strain effects, improving the biomechanical bone tissue modeling process [52,53].

von Mises failure criteria have been established in the literature to adequately represent the complex stress pattern behavior in dental implant–biological structure interaction. Finite element von Mises stress results under occlusal loading indicated that the three-piece implant system has a normal stress distribution pattern of 131–175 MPa, mainly presented in a micro-thread design with a stress peak value of 395.53 MPa under a micro-thread. The platform switching design in the implant body indicated the prevention of crystal bone loss, with maximum stress values of 1.48 and 1.36 MPa (oblique and occlusal loading, respectively) in bony tissue; peak stress values and distribution fields along biological structures are consistent with the findings of different research groups evaluating normal trabecular and cortical tissues [51,52,54,55]. During oblique loading, a much higher peak value is reached, corresponding to 481.16 MPa. This value can be attributed to the complex conditions that induced greater stress and strain in the implant–bone interface compared with axial loading; this is in line with the notable increment in stress distribution reported in common titanium three-piece implant systems [56,57,58,59]; nevertheless, the yield stress limit has not been exceeded, yet it represents a susceptible zone close to failing.

The two-piece implant system exhibited a typical stress distribution pattern under occlusal loading, with principal stresses ranging from 65.934 to 58.626 MPa being predominantly concentrated across the abutment height and the upper part of the implant body. Notably, peak stress values ranging between 36.63 and 29.304 MPa were observed at the groove of the abutment (due to a cross-section change) and the implant body’s shoulder height (right above micro-threads). Despite geometric design differences, zirconia dental implants have similar stress peak values and distribution in bony structures, as reported in the literature, exhibiting a range of values ranging between 20 and 100 MPa, respectively, under similar loads [45,60]. The stress distribution pattern within the surrounding biological tissues was concentrated in the cervical region of the cortical bone under both loading conditions employed, with peak stress fields accumulating in the alveolar zone (58.346 and 44.714 MPa for occlusal and oblique loads, respectively), which can be attributed to zirconia implant designs; this effect is in agreement with the findings provided by a different investigation group reporting more distributed and even stress concentrations in the alveolar region for two-implant systems [61,62,63].

The effects generated by applying both loading conditions triggered the highest stress concentration patterns at the neck (above micro-threads) and the abutment union with the implant body regions in the single-piece implant system. These numerical results are concordant with previous studies, reinforcing the robustness and reliability of our research. They suggest that these areas are of particular attention, especially in the design of one-piece implants, particularly those with reduced diameters in the implant body–abutment union. In addition, a stress peak concentration in the cervical region of cortical tissue was observed, along with uniform low-stress concentration fields within the bone structures [14,61,64].

The analysis of the strains and deformations can be considered essential in predicting the behavior of implant–abutment interaction, micromotion between elements, and bone resorption (implant–biological tissue interaction). Therefore, the employment of the finite element method is advantageous in providing relevant data related to micromotion during the normal chewing process as experimental methods are inaccessible for acquiring information. The one-piece implant system exhibited lower displacement and strain values, having maximum values of 0.0039 mm (occlusal force), 0.0099 mm (oblique force), and 0.6 μm in the implant polished collar (axial and angled loading conditions), and 13.8 μm in cortical and trabecular bones. Meanwhile, maximum displacements of 0.0029 and 0.0071 mm were acquired in the two-implant configuration in the two types of loading conditions, respectively; the strain distribution in this implant was reported with 0.3 and 0.47 μm peak values at the joint of the abutment–implant body in axial and angled force applications, along with a average distribution of 0.21–0.25 μm for occlusal and 0.26–0.31 μm for oblique loading in this same region. Peak values of 9.7 and 10.3 μm were found in bony tissues. Finally, the three-piece implant presented peak deformations of 0.0088 and 0.0342 mm, suggesting a tendency to micromovements due to its piece’s joints; moreover, there was a maximum strain distribution of 6.5 and 7.8 μm for the axial and inclined loading conditions. Uniform strain distribution, mainly a green spectrum in the isochromatic scale, with values ranging between 0.36–0.43 μm and 0.43–0.52 μm, corresponding to the general average values along the implant–abutment interaction. On the other hand, this implant system displayed 16.4 and 16.9 μm in cortical and cancellous structures. These results align with the threshold reported by other researchers when evaluating stress distribution, assuming ideal osseointegration conditions [65,66,67].

Analyzing the three implant systems’ commercially available options provided relevant data on the performance of different implant viable options. It is relevant to point out the superior performance of the zirconia two-implant system, having slightly higher strain and deformation peak values than the single-unit implant; nevertheless, it presents a better stress distribution than the other two implants. The titanium three-piece implant presented an outstanding stress distribution with minimum peak values in the biological tissues, mainly attributed to the material and geometric design (morse taper connection) and thread design (reverse buttress and narrower thread pitch) [49]. As expected, this implant system had higher strain/displacement range values; therefore, mechanical stress in this implant was close to exceeding yield strength, which could also be associated with micromovements leading to partial or poor osseointegration.

The main aim of this study was to biomechanically compare different implant systems with feasible results that aligned reasonably well with previously published data to provide a wider perspective on the implant–biological interaction behavior for the selection criteria. Although the methods presented are notable in the implant behavior prediction, the final decision in the implant selection process must depend on a clinical assessment of specific patients’ needs for implant placement [1,7,10,68]. Furthermore, based on the results obtained, a significant approach from the research was to generate valuable insights into the design of personalized dental implants according to piece number, geometric, and material considerations.

While the current research provided valuable insights, certain limitations inherent to the study have to be acknowledged. The influence of the cement layer was not considered due to the assumption of perfect connection to avoid the use of any screw element. Moreover, ideal osseointegration was assumed, and the analysis was conducted under the simplification of static occlusal and oblique loading conditions. Consequently, bone tissue orthotropic properties could be better represented directly from computer tomography intensity data and morphology of the jaw molar region. Additionally, the numerical results presented in this study should be interpreted as relative values within the scope of the specified design parameters. Future research should investigate a broader range of implant configurations and loading conditions to understand the impact of implant design variations on stress distribution.

## 5. Conclusions

The current paper demonstrates the feasibility of utilizing numerical simulations to predict the complex biomechanical behavior of different implant systems under the same conditions to deepen the knowledge of biological effects triggered within the scope and limitations of the finite element models. Therefore, it heightens the substantial value of numerical methods in analyzing challenging biomechanical systems, particularly in dental implantology. Numerical results notably reinforce the use of ceramic biomaterials, demonstrating comparable osseointegration and clinical success rates compared with titanium implants. It was observed that zirconia exhibited greater energy absorption capacity compared with titanium, resulting in reduced overall deformations under load. Consequently, the findings of this study demonstrated that the two-implant system is the best viable option due to its outstanding balance performance of the implant system by itself and the surrounding biological tissues’ stress and strain behavior, generating fewer micro-strains than titanium three-piece systems to avoid any micro-gaping that could lead to bone resorption. In addition, the two-implant system had better stress distribution and was not as susceptible to mechanical failure as the single implant. Therefore, the relevant data is in accordance with recent clinical trends of ceramic two-implant systems being more frequently used. Furthermore, in clinical practice, changing a two-piece system is less of a complicated replacement process in the case of failure than a one-piece system. Finally, the results reported in this research need additional clinical validation before definitive conclusions can be drawn regarding the implications of the findings presented in this study; nonetheless, it encourages generating innovative solutions to enhance the selection criteria and maximize patient success rates through the convergence of multiple tools and scientific fields.

## Figures and Tables

**Table 1:** Total number of elements and nodes for each numerical analysis.

Implant Systems	Nodes	Elements
Three-piece system	812,693	527,194
Two-piece system	1,785,273	1,249,596
One-piece system	2,392,206	1,698,241

**Table 2:** Mechanical properties of the materials employed [44,45,46,47,48,49,50,51].

Material	Elastic Modulus (MPa)	Shear Modulus (MPa)	Poisson’s Ratio (ν)	Yield Strength (MPa)	Density (g/cm^3^)
Cortical bone [44,45,46]	Exx 9600	Gxy 3100	νxy 0.55	115	1.8
	Eyy 9600	Gyz 3510	νyz 0.3		
	Ezz 17,800	Gxz 3510	νxz 0.3		
Trabecular bone [44,45,46]	Exx 144	Gxy 53	νxy 0.23	32.4	1.2
	Eyy 99	Gyz 63	νyz 0.11		
	Ezz 344	Gxz 45	νxz 0.13		
Gingival [51] tissue	19.6	7.5385	0.30	-	-
Titanium grade 4 [47,48]	104,000	39,394	0.32	482	4.54
Zirconia [49]	210,000	80,769	0.3	1050	6.08
Yttria-stabilized zirconia [50]	210,000	80,769	0.3	1200	6.05

## Data Availability

All data generated or analyzed during this study are included within the article.

## Appendix A

**Table A1:** Three-piece dental implant system summary of numerical evaluation results for both loading conditions analyzed.

Type of Analysis	Dental Implant System				Biological Structures			
	Occlusal Loading		Oblique Loading		Occlusal Loading		Oblique Loading	
	Maximum Value	Minimum Value	Maximum Value	Minimum Value	Maximum Value	Minimum Value	Maximum Value	Minimum Value
Total elastic strain (mm/mm)	0.0065	1.8568 × 10^−6^	0.0078	1.4031 × 10^−6^	0.0164	6.111 × 10^−9^	0.0169	4.2341 × 10^−9^
Elastic strain X axis (mm/mm)	0.00092	−0.0020	0.0013	−0.0031	0.0024	−0.0042	0.0045	−0.0077
Elastic strain Y axis (mm/mm)	0.0013	−0.0024	0.0030	−0.0038	0.0159	−0.0173	0.0133	−0.0138
Elastic strain Z axis (mm/mm)	0.0017	−0.0023	0.0014	−0.0017	0.0043	−0.0072	0.0016	−0.0033
Nominal stress X axis (MPa)	142.99	−350	188.97	−544.16	0.5361	−0.1716	0.6907	−0.4608
Nominal stress Y axis (MPa)	171.38	−419.97	374.89	−505.54	1.0772	−0.4997	0.6025	−0.3345
Nominal stress Z axis (MPa)	208.79	−458.78	172.95	−364.14	1.0909	−0.5488	0.6252	−0.3747
Shear stress XY plane (MPa)	141.67	−109.96	248.79	−92.824	0.5677	−0.3134	0.7578	−0.2879
Shear stress YZ plane (MPa)	127.4	−202.93	169.67	−158.61	0.7375	−0.5781	0.3521	−0.4080
Shear stress XZ plane (MPa)	54.801	−92.053	95.652	−102.84	0.0762	−0.1086	0.4496	−0.3929
von Mises stress (MPa)	359.53	6.1561 × 10^−8^	481.16	4.3692 × 10^−8^	1.4802	5.8888 × 10^−5^	1.3674	4.3552 × 10^−5^

**Table A2:** Two-piece dental implant system summary of numerical evaluation results for both loading conditions analyzed.

Type of Analysis	Dental Implant System				Biological Structures			
	Occlusal Loading		Oblique Loading		Occlusal Loading		Oblique Loading	
	Maximum Value	Minimum Value	Maximum Value	Minimum Value	Maximum Value	Minimum Value	Maximum Value	Minimum Value
Total elastic strain (mm/mm)	0.00035	9.9409 × 10^−7^	0.00047	1.6882 × 10^−6^	0.0097	5.6035 × 10^−9^	0.0103	4.1998 × 10^−9^
Elastic strain X axis (mm/mm)	0.00011	−8.2692 × 10^−5^	0.00018	−0.00019	0.0015	−0.00091	0.0059	−0.0055
Elastic strain Y axis (mm/mm)	0.00031	−0.00030	0.00024	−0.00041	0.0020	−0.0061	0.0013	−0.0031
Elastic strain Z axis (mm/mm)	0.00010	−0.00017	0.00044	−0.00029	0.0028	−0.0022	0.0011	−0.00099
Nominal stress X axis (MPa)	28.877	−33.658	44.85	−73.89	15.692	−17.858	16.876	−18.66
Nominal stress Y axis (MPa)	73.15	−74.468	62.198	−106.43	15.607	−17.845	10.792	−14.251
Nominal stress Z axis (MPa)	37.056	−62.328	90.195	−66.541	23.193	−26.748	15.309	−17.654
Shear stress XY plane (MPa)	18.189	−15.951	36.991	−16.948	8.8606	−5.9468	11.554	−2.767
Shear stress YZ plane (MPa)	35.921	−27.175	27.292	−22.803	33.292	−25.354	16.687	−13.238
Shear stress XZ plane (MPa)	9.2771	−8.7786	34.829	−35.015	2.8092	−2.7919	14.867	−19.372
von Mises stress (MPa)	65.934	4.5526 × 10^−8^	99.037	3.0295 × 10^−8^	58.346	4.5526 × 10^−8^	44.714	3.0295 × 10^−8^

**Table A3:** One-piece dental implant system summary of numerical evaluation results for both loading conditions analyzed.

Type of Analysis	Dental Implant System				Biological Structures			
	Occlusal Loading		Oblique Loading		Occlusal Loading		Oblique Loading	
	Maximum Value	Minimum Value	Maximum Value	Minimum Value	Maximum Value	Minimum Value	Maximum Value	Minimum Value
Total elastic strain (mm/mm)	0.00068	6.672 × 10^−7^	0.00061	7.0831 × 10^−7^	0.0138	5.2261 × 10^−8^	0.0137	3.8298 × 10^−8^
Elastic strain X axis (mm/mm)	9.7492 × 10^−5^	−0.00010	0.00019	−0.00021	0.0017	−0.0052	0.0107	−0.0063
Elastic strain Y axis (mm/mm)	0.00044	−0.00029	0.00045	−0.00057	0.0035	−0.0069	0.0035	−0.0050
Elastic strain Z axis (mm/mm)	0.00020	−0.00024	0.00011	−0.00012	0.0039	−0.0076	0.0015	−0.0025
Nominal stress X axis (MPa)	54.761	−91.952	42.276	−80.982	54.761	−91.952	41.876	−80.982
Nominal stress Y axis (MPa)	117.78	−95.423	104.7	−131.59	45.381	−95.423	38.068	−81.115
Nominal stress Z axis (MPa)	96.615	−128.27	41.318	−95.758	96.615	−128.27	41.318	−95.758
Shear stress XY plane (MPa)	21.358	−19.268	55.732	−24.949	21.358	−18.967	27.574	−3.6452
Shear stress YZ plane (MPa)	64.723	−41.365	26.792	−27.304	43.961	−38.709	26.792	−27.304
Shear stress XZ plane (MPa)	11.45	−12.286	21.199	−17.979	10.813	−10.687	22.491	−22.362
von Mises stress (MPa)	136.43	7.0043 × 10^−7^	128.74	4.1083 × 10^−7^	83.472	7.0043 × 10^−7^	66.938	4.1083 × 10^−7^
